# Application of MALDI-TOF-MS in the surveillance of microbial diversity in butter production: a case study of Polish dairy

**DOI:** 10.1038/s41598-026-43570-0

**Published:** 2026-03-11

**Authors:** Ewelina Sibińska, Iwona Adamczyk, Agnieszka Ludwiczak, Michał Złoch, Paweł Pomastowski

**Affiliations:** 1 Institute Of Advanced Studies, Centre for Modern Interdisciplinary Technologies, Nicolaus Copernicus University in Toruń, Wileńska 4 Str., 87-100 Toruń, Poland; 2 Department of Physiology and Toxicology, Kazimierz Wielki University, Chodkiewicza 30 Str., Bydgoszcz, Poland; 3 Department of Immunology, Faculty of Biological and Veterinary Sciences, Nicolaus Copernicus University in Toruń, Lwowska 1 Str., 87-100 Toruń, Poland

**Keywords:** MALDI, Microbiota, Bovine milk, Butter production, Biological techniques, Biotechnology, Microbiology

## Abstract

The primary objective of the study was to adapt and evaluate the utility of the rapid MALDI-TOF MS (Matrix-Assisted Laser Desorption/Ionization Time-of-Flight Mass Spectrometry) identification method for comprehensive microbiological safety monitoring across the farm-to-butter chain. The research was conducted as a detailed case analysis within a selected Polish dairy facility, focusing on the microbial profiling from the milking to the final product stage. The application of this method enabled comprehensive microbiota profiling of 400 milk samples, along with 63 additional samples from nine stages of the butter production process, resulting in the identification of 146 different cultivable microorganisms. The microbiota of fresh bovine milk was primarily composed of bacteria from the genera *Staphylococcus*, *Corynebacterium*, *Aerococcus*, and the species *Bacillus licheniformis*. The highest microbial diversity was observed in samples taken from tankers transporting milk to the dairy. Pasteurization effectively reduced the total number of microorganisms, but did not completely eliminate spore-forming or psychrophilic bacteria. The results emphasize the need to maintain a high level of hygiene at every stage of production in order to ensure microbiological safety and quality of dairy products.

**Supplementary Information:**

The online version contains supplementary material available at 10.1038/s41598-026-43570-0.

## Introduction

Milk and dairy-derived foods serves as an ideal nutrient broth for the proliferation of numerous foodborne microorganisms. Milk obtained from healthy mammary glands, at the point of milking, is theoretically expected to be safe for human consumption. However, once the milk is secreted, it becomes highly susceptible to contamination from various sources, including the animal’s skin, feces, barn air, milking equipment, storage containers, and the surrounding environment^1–3^. Pathogens like *Salmonella*, *Listeria*, *Escherichia*, and *Bacillus* species can easily contaminate the milk during the milking/production process, thereby creating a significant risk of foodborne illnesses. Moreover, should be noted that subclinical infections in cows can result in direct excretion of pathogens into the milk. *Salmonella*,* Listeria*,* Brucella spp*., or *Mycobacterium bovis* can be present in milk without any visible signs of mastitis or illness in the animal. All this can lead to outbreaks of infections, especially in vulnerable populations like elderly, children or with weakened immune systems^4,5^.

Therefore, safety and production are closely interconnected throughout the entire dairy food chain, from good agricultural practice, through production and processing to final consumption. At each stage, various methods prevent contamination and ensure product safety. In farming, proper animal care, hygiene, and disease control minimize pathogens, while clean milking equipment and a healthy environment reduce microbial contamination^6^.

The primary responsibility for ensuring the safety of dairy products falls on dairy processors, who implement a variety of methods to control contamination during production. These methods mainly include thermization or pasteurization, which effectively eliminates harmful pathogens by heating the milk to a specific temperature. Bactofugation, a process that involves the removal of harmful bacteria from milk through centrifugal force, is also employed to improve product safety^7^. Additionally, modern dairy plants often use technologies like ultrafiltration and microfiltration to further reduce microbial loads and improve the quality of milk and dairy products^8^. Regardless of the method employed by dairies, all microorganisms should be effectively eliminated. However, in cases of insufficient hygiene or inadequate sealing of the processing system, secondary contamination may still occur.

Although butter has a high milk fat content (> 80%), which may reduce the risk of microbial contamination compared to other dairy products, this does not mean it is free from contamination. The fat in butter creates a certain barrier that hinders the growth of many microorganisms, especially compared to more moist dairy products such as milk or yogurt^9^. Butter production begins with the separation of cream by centrifugation. The cream is then filtered and pasteurized, typically at 72–76 °C for 15–20 s. The next step is cooling to 8–12 °C, which initiates the crystallization of milk fat, allowing the formation of the appropriate fat structure. During churning, fat globules stick together, which leads to the formation of butter grains. The remaining liquid, i.e. buttermilk, is drained, and the butter is transformed into a continuous fat phase with a finely dispersed water phase. The finished butter is packaged and stored under controlled refrigeration (typically 4–6 °C) to limit microbial growth^10,11^. However, contamination may occur at any stage, especially since the dairy production system is complex and contains hard-to-clean areas - such as valves, shafts, and seals - that can harbor bacterial biofilms causing milk spoilage^12^. The same is true for milking equipment. Therefore, stringent microbiological cleanliness control is essential throughout the entire production chain to ensure the safety of dairy products.

This study aimed to implement a cutting-edge Matrix-Assisted Laser Desorption/Ionization Time-of-Flight Mass Spectrometry (MALDI-TOF MS) technique, renowned for its rapid and highly accurate identification of cultivable bacterial and fungal species based on their unique protein profiles. The method relies on ionization of microbial proteins by a laser beam in the presence of a matrix and subsequent separation of ions according to their mass-to-charge ratio during time-of-flight analysis, generating species-specific spectral fingerprints^13^. Using this approach, we comprehensively analyzed the microbial dynamics throughout the bovine milk butter production chain, from the farm to the final product in a selected dairy plant in Poland. This advanced approach allowed for precise microbiological tracking, serving as a reliable tool to assess shifts in the microbial community and pinpoint critical points within the production chain. Selected isolates were also analyzed by 16 S rRNA sequencing (often considered the reference method for MALDI^14^ to support the reliability of the implemented approach.

## Methodology

### Samples

The research material consisted of samples of bovine milk and samples from various stages of butter production.

Milk: samples were collected from the farm Agrofarm Sp. z o.o. Jurkowice Pierwsze, 82–410 Stary Targ (Poland). The herd of cows consisted of approximately 370 adult females. The herd was housed year-round in a tie-stall barn with a concrete floor covered with straw bedding. Their diet consisted exclusively of haylage, and water was supplied by an automatic watering system.

Milk samples were collected from 50 random cows, for each cow from fore and hind udders separately. Before collection, the udders were washed, disinfected by immersion in an iodine-based fluid for washing udders and teats before milking, and dried, and the first streams of milk were removed. Samples were collected in sterile 50 mL falcons. The collected material was stored at 5–8 °C and transported to in Centre for Modern Interdisciplinary Technologies Toruń (Poland), where microbiological cultures were immediately performed.

*Butter production*: sampling was carried out at different stages of butter production in the Polmlek Grudziądz dairy: a cold store on the farm (*n* = 9), a tanker transporting milk from the farm to the dairy (*n* = 22), tanks in dairy (*n* = 4), cream before pasteurization (*n* = 4), cream after pasteurization (*n* = 4), cream before crystallization (*n* = 4), cream after crystallization (*n* = 4), buttermilk (*n* = 4) and butter (*n* = 8).

All samples were collected in four repetitions between March and April 2024. In total, milk was collected from 200 cows. Due to the separate collection of milk from the fore and hind udders of each cow, cultures and breeding were carried out in accordance with this division. A total of 400 milk samples were tested (200 each from the fore and hind udders).

During the butter production process, samples were collected at nine key technological stages, allowing for the monitoring of changes in raw material and product parameters during subsequent processing phases. The number of samples taken varied – in some cases, only one was taken when the process being examined occurred in a single element of the production line, while in other situations, such as during milk transport by truck, larger samples were taken because the entire product could not fit in a single test unit.

### Isolation of microorganisms

To prepare the inoculum, 1 mL of liquid samples (milk, cream, buttermilk) or 1 g of solid samples (butter) was aseptically transferred into a sterile 15 mL falcon tube containing 9 mL of sterile peptone water (Sigma Aldrich, Germany). In the case of solid samples, the diluent was pre-warmed to 40 °C prior to addition. The mixtures were homogenized by vortexing for 30–60 s. For fatty or hard samples, vortexing was continued until a uniform suspension was achieved. All samples were plated in a series of dilutions in peptone water (Sigma Aldrich, Germany) from 10^− 1^ to 10^− 3^ on solid media set: TSA (Tryptic Soy Agar, Sigma Aldrich, Germany)—universal medium, DRBC (Dichloran Rose-Bengal Chloramphenicol Agar Base, Oxoid, UK) - selective medium for the isolation of yeasts and molds that are important in food spoilage, and, SCH (Scheadle Agar, Sigma Aldrich, Germany)—for culturing anaerobic bacteria, MRS (De Man–Rogosa–Sharpe Agar, Sigma Aldrich, Germany)—selective medium for isolation of Lactic Acid Bacteria (LAB).

The prepared media with the inoculated material were incubated: TSA media in aerobic conditions at 37 °C for 48 h; SCH and MRS media in anaerobic conditions at 30 °C for 72 h, obtained with the use of containers and anaerobic condition generators (GenBox Anaer, bioMérieux, France); DRBC media in aerobic conditions at 30 °C for 72 h. From the grown cultures, single colonies were selected based on morphological differences and subcultures were prepared on the same media to obtain pure cultures.

### Cultivable microbiota identification

MALDI-TOF Mass Spectrometry: Identification of isolated strains was performed using a MALDI mass spectrometer EXS2600, Zybio Inc. China by direct on-plate extraction – sample preparation method suitable for both Gram type of bacteria and demonstrated short handling time. For this purpose, the isolated colony was spread as a thin layer directly on the sample position. Then, 1 µl of 70% formic acid was applied to the sample, and after it had dried, 1 µl of HCCA matrix solution (concentration of 10 mg/ml solution containing 50% acetonitrile, 47.5% water and 2.5% trifluoroacetic acid) was applied. The collected spectra were analyzed using the Ex-Accuspec ver. V1. Bacterial Test Standard (Bruker, Germany) was used as a calibrator. Identification was based on the score values generated by the system, where a score ≥ 2.0 was considered reliable for species-level identification, scores between 1.7 and 1.99 indicated genus-level identification, and scores < 1.7 were regarded as unreliable. In total, 6129 microbial colonies were analyzed by MALDI-TOF MS, corresponding to an average of 13.2 colonies identified per sample. For statistical analyses, only the highest-scoring MALDI-TOF MS identification result per species and sample was retained. Accordingly, the number of isolates reported in the study (*n* = 2179) represents non-duplicated, best-quality identifications.

*16 S rRNA*: Representative strains (to confirm MALDI identification) and any strains not identified by the MALDI method were selected for further analysis. The total bacterial genomic DNA isolation was performed using the E.Z.N.A.^®^ Bacterial DNA Kit (Omega Bio-tek, USA) and overnight cultures grown on TSA plates (Sigma-Aldrich, Germany) at 37 °C. The extracted DNA was used for the 16 S rRNA gene amplification via polymerase chain reaction (PCR) technique using the universal bacterial primers 1492R (5’-TACGGYTACCTTGTTACGACTT-3’) and 27 F (5’-AGAGTTTGATCMTGGCTCAG-3’), thermostable TaqDNA polymerase (Qiagen, Hilden, Germany), Mastercycler PRO S thermocycler (Eppendorf AG, Hamburg, Germany), and PCR program established in the earlier work^15^. Sequencing was carried out in Genomed (Warsaw, Poland) using the Sanger dideoxy method using the same primers, contigs were assembled via BioEdit Sequences Alignment Editor ver. 7.2.5^16^, and consensus sequences were compared with references sequences in rRNA/ITS databases of the National Center for Biotechnology Information via the BLAST algorithm (https://blast.ncbi.nlm.nih.gov/Blast.cgi? PAGE_ TYPE=Blast Search). Criterion for species-level confidence of identification was assumed: (i) ≥ 98.7% identity with the reference sequences in rRNA/ITS databases for bacteria demonstrated high 16 S intra-genus variability; (ii) ≥ 99.0% identity threshold for bacteria demonstrated high similarity within 16 S region (e.g., *Escherichia*,* Bacillus subtilis* complex, *Citrobacter freundii* complex) and ≥ 0.5% variation in percentage of identity with reference database between the best matches.

### Statystical analysis

Statistical analyses and visualizations were performed using the Python programming environment (version 3.9). Data analysis was conducted with the Pandas library (version 2.2.3), while graphical representations were generated using Matplotlib (version 3.7.4). A Chi-Square test was applied to determine whether the difference in the number of identified strain and genera between collection times, as well as between the front and hind udder, was statistically significant. Additionally, the Chi-Square test was used to evaluate the effects of the milk processing stage and sample collection time on the type of identified genera. The Wilcoxon signed-rank test was performed to assess whether the difference in the number of identified species between the front and hind udder was statistically significant. The Kruskal-Wallis H test was applied to examine differences in genus distribution across different milk processing stages. As a post hoc analysis, the pairwise Mann-Whitney U test with Bonferroni correction was conducted to identify significant differences in species composition between milk collection times.

To assess changes in biodiversity across different milk processing stages, several diversity indices were analyzed. Species Richness (S) was determined for each processing stage. Each milk processing stage was treated as a separate analytical sample group. For each stage, the indices were calculated based on the number of isolates of each species and the total number of isolates obtained at that stage, as summarized in Table S1 (Supplementary Materials). The Shannon-Wiener index (H’) was calculated to measure the uncertainty associated with predicting the occurrence of a specific species within a given set of species and species evenness according to the formula: H’=−∑[(pi) ×log (pi)], where:

pi—the proportion of individual species identified at a given milk processing stage in the total number of all species obtained at that stage,

pi = n/N, where:

n—is the number of isolates of the species identified at a given milk processing stage,

N—the total number of all species obtained at that stage.

Simpson’s Diversity Index (1–D) was calculated as a measure of diversity, taking into account the number of isolates of the species and the relative abundance of each species at the given processing stage, according to the following formula:

$${\mathrm{D}} = \frac{{\sum n~\left( {n - 1} \right)}}{{N~\left( {N - 1} \right)}}$$

The evenness of a community was presented using Pielou’s Evenness Index (J’) and calculated according to the following formula:

$${\mathrm{J}}^{\prime } = \frac{{H^{\prime } }}{{log2S}}$$

To facilitate the comparison of indices on a single graph, min-max scaling was applied, normalizing each index value to a range of 0–1.

## Results

### MALDI and 16 S rRNA in microbiota identification

All cultivable microorganisms isolated from milk and samples collected during the butter production process were rapidly identified using the MALDI technique. A total of 2179 isolates were identified, of which 85.0% (*N* = 1852) were classified at the species level (score value ≥ 2.00). The average identification value was 2.02 ± 0.21. The application of MALDI system enabled the identification of 139 bacterial species across 60 genera, as well as ten fungi species, mostly yeast from the genera *Candida* and *Kluyveromyces*. Among the identified bacteria species, the majority were Gram-positive, accounting for 68%.

For selected strains, especially those representing various genera, frequently occurring species, or isolates unidentifiable (log score < 1.7) via MALDI, additional molecular identification based on the 16 S rRNA gene was performed to confirm species classification and resolve insufficient MALDI identification (Supplementary Table S2). The application of a secondary identification method enabled the confirmation of the results obtained using the MALDI technique in the vast majority of cases and provided more precise identification in a few instances. The use of 16S rRNA gene sequencing was insufficient for the precise identification of most bacteria belonging to the genus *Bacillus* and several closely related strains within the genera *Staphylococcus* and *Streptococcus*. Consequently, identification was limited to the group or complex level (e.g., *Bacillus cereus* group, *Streptococcus lutetiensis/equinus* group) and is presented as such in this article. Supplementary Table S3 provides a comprehensive list of all microorganisms identified in the tested samples.

### Raw bovine milk microbiota

A total of eight fungi and 108 bacterial species were identified in raw milk, representing a diverse microbiota composed of various genera. The highest bacterial diversity was observed within the genera *Corynebacterium*, *Staphylococcus*, and *Streptococcus*, with 19, 16, and 11 different species identified, respectively. Milk samples contained up to 15 different microbial species, with an average of 7.5 species per sample. The microbiota was predominantly composed of Gram-positive bacteria, which accounted for 92.7% of the isolates. All samples contained at least one Gram-positive bacterium, and in 64% of the samples, the microbiota consisted exclusively of Gram-positive species. Additionally, Gram-negative bacteria and fungi were present in 26% and 6% of the samples, respectively. Both bacterial types and fungi, was observed in only 5% of the samples.

**Fig. 1:**
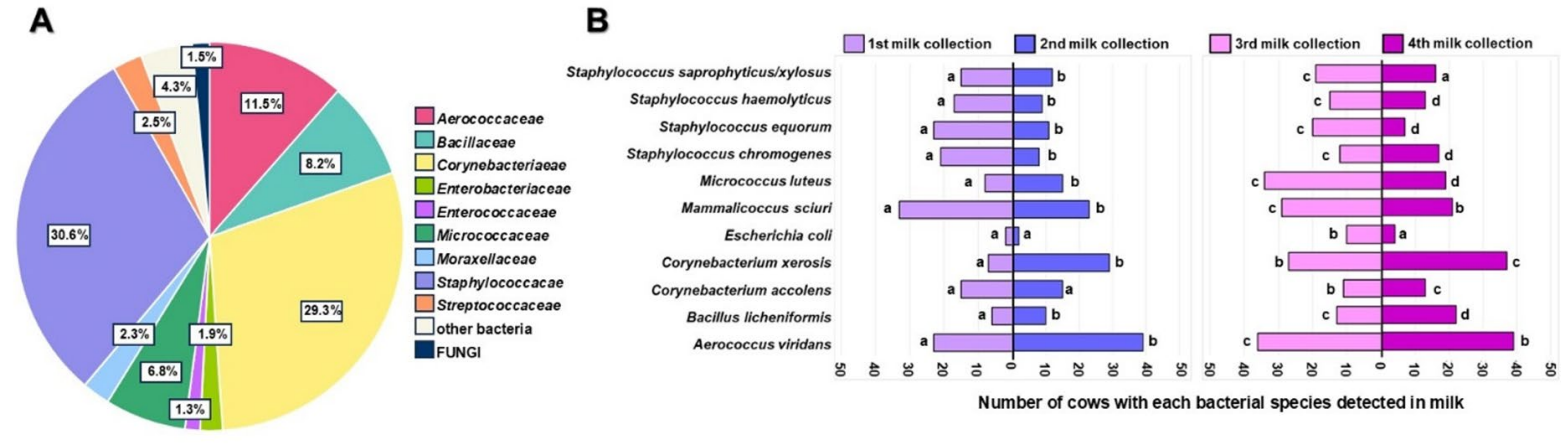
(**A**) Percentage of microorganism identification in bovine milk within given families. (**B**) Analysis of the frequency of occurrence of the 10 most frequently identified species (and *E. coli*) in four different milk collection times. Significant differences in species occurrence across the collection times are indicated by different letters.

The most abundant families in fresh milk were *Staphylococcaceae* and *Corynebacteriaceae*, accounting for 30.6% and 29.3% of the total identified microorganisms, respectively (Fig. 1A). The ten most frequently isolated bacterial species (and *Escherichia coli*, as an indicator of fecal contamination and a potential risk to consumer health) in milk are shown in Fig. 1B, with their occurrence in samples categorized across four collection time points over two months (March–April 2024). The Chi-square test confirmed a statistically significant difference in species occurrence across the four milk collection time points (*p* < 0.001). Moreover, the species composition varied significantly between the four collection of milk (*p* < 0.001) for most of the identified species. A significantly higher abundance of *Staphylococci*, including *S. haemolyticus*, *S. equorum*, *S. chromogenes*, and *Mammaliicoccus sciuri*, was observed during the first stage of milk collection compared to the subsequent milk collection stages. On the other hand, *Aerococcus viridans*, and *Corynebacterium xerosis*, was significantly more frequent in the subsequent samplings.

**Fig. 2:**
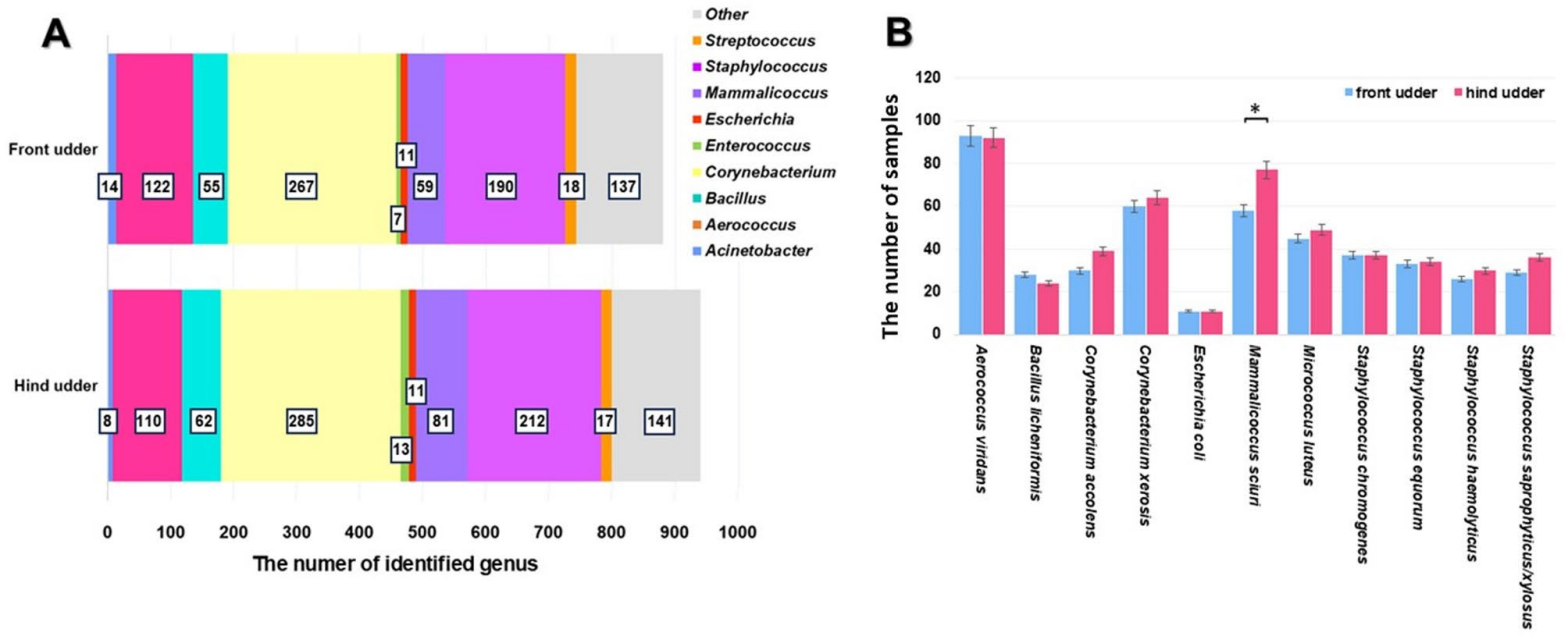
Identified microbial genera (**A**) and most abundant species (**B**) categorized by front and hind parts of the udder. The numbers in the rectangles (in Fig. **A**) indicate how many times a given genus was identified in milk samples from the front and rear udder quarters, respectively. Error bars (in Fig. **B**) represent the 95% confidence interval. Asterisks indicate statistically significant differences at *p* ≤ 0.05.

A total of 56 genera were identified in the milk from front udder and 55 in the milk from hind udder. Among all identified genera 9 were detected with a relative abundance exceeding 1% in both the front and hind udders (Fig. 2A), and their distribution did not show statistically significant differences between the two regions (*p* = 0.924). The most abundant genus was *Corynebacterium (N* = 267 and 285 in the front and hind udders, respectively), followed by *Staphylococcus* (*N* = 190 and 212 in the front and hind udders, respectively). Although *Enterococcus*, *Acinetobacter*, and *Escherichia* ranked among the ten most frequently isolated genera overall, strains belonging to these genera were detected in the fewest analyzed samples (less than 14 samples from front and hind udder). Among the genera with a relative abundance below 1%, some genera - including *Brevibacterium*,* Delftia*,* Glutamicibacter*,* Klebsiella*,* Kluyveromyces*,* Naganishia*,* Pantoea*,* Streptomyces*, and *Paenibacillus* – were detected exclusively in the front udder and were absent from the hind udder. The number of *M. sciuri* differed significant between the front and hind udders (*p* = 0.016, Fig. 2B). General trends in the distribution of bacterial genera were observed across all four sampling points.

The occurrence of LAB strains, such as *Lactobacillus* and *Lactococcus*, was limited, observed in only 7 of the 200 tested bovine milk samples, and more often in the front udders. Considered as pathogenic bacterial genera, including *Acinetobacter*, *Escherichia*, Proteus, *Pseudomonas*, and *Raoultella* were detected in 35 of the analyzed bovine udder samples, with their presence observed in both front and hind quarters. Furthermore, in cases where a higher initial diversity of this genera was observed, these microorganisms tended to persist, being detected at two or more of the four sampling time points. Notably, fungal organisms were also identified in these samples. The opportunistic pathogen *M. sciuri* was also detected in two-thirds of the samples positive for other pathogenic genera.

### Butter production intermediates

**Fig. 3:**
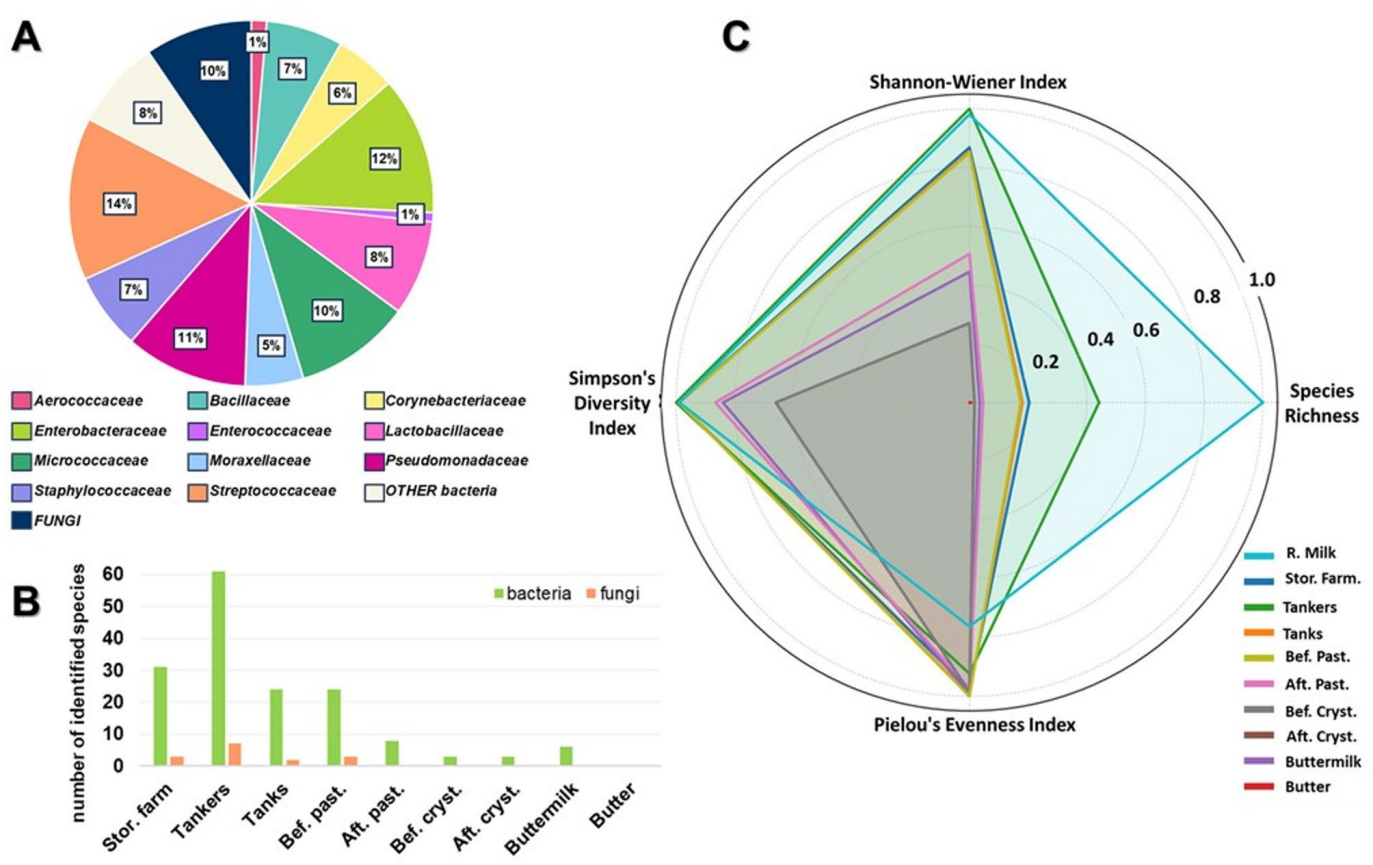
(**A**) Percentage of individual families identified in butter production samples. (**B**) microbiota richness of the analyzed samples at different stages of butter production. (**C**) Biodiversity indices across analyzed milk processing stages. See Fig. 3 for the explanation of abbreviations. R. Milk- raw milk, Tankers - cold store at the farmer’s, Tank - storage in a tank at the dairy, Bef.Past- before pasteurization, After.Past- after pasteurization, Bef.Cryst- before crystallization, After.Cryst- after crystallization.

Across the butter production continuum, from farm cold storage through transport to the dairy facility, a total of 79 bacterial species and 9 fungal species were identified. This included the detection of 34 species that represent novel additions to the microbial profile compared to the fresh bovine milk. The most frequently identified bacteria in the samples were *Streptococcaceae*, *Enterobacteriaceae*,* Pseudomonadaceae*, and *Micrococcaceae* (Fig. 3A), which together accounted for nearly half (47%) of the identified isolates. The greatest diversity in the microbiota of the samples was observed during the storage and transport stage (Fig. 3B), specifically while the milk was being transferred from the farm to the dairy in tankers (61 species of bacteria and 7 species of fungi). Fungal species were identified mostly in the early stages. Microbial diversity decreased significantly after pasteurization, with no species detected in the final product – butter, whereas six bacterial species were still identified in buttermilk. The highest values of species richness and the Shannon-Wiener index were recorded for the initial raw material and early milk storage stages (Fig. 3C), specifically in raw milk, tankers, and cold storage at the farm (S = 172, 76, and 35, respectively). Each stage of milk processing caused biodiversity to systematically decline (from raw milk to butter). In raw milk, microbial populations were more diverse, as reflected by the relatively high Shannon–Wiener index values (3.89 for tankers and 3.33 for tanks). However, pasteurization had the strongest impact, significantly reducing both species richness and evenness, leading to a 10-fold and 2-fold decrease in the S and H’ indices, respectively.

**Fig. 4:**
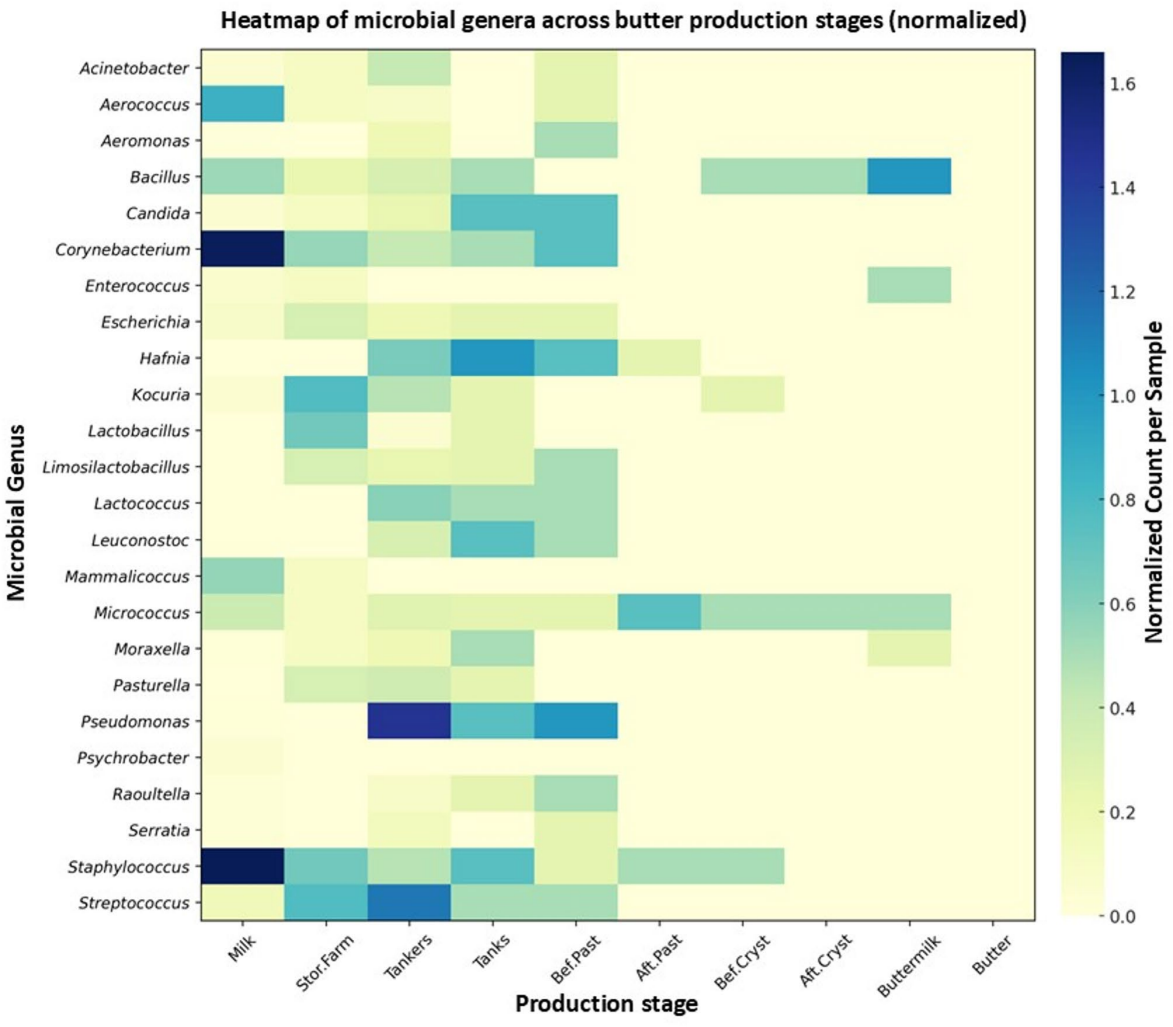
– Heatmap of the distribution of identified microbial genera across butter production stages (normalized to the number of samples collected at each stage).

The relative abundance of identified genera occurring with a frequency above 1% varies across different stages of milk transportation and processing (Fig. 4). In raw milk, the most abundant genera were *Corynebacterium* (*N* = 329), *Aerococcus* (*N* = 170), and *Staphylococcus* (*N* = 332), as well as *Bacillus* (*N* = 107) and *Mammalicoccus* (*N* = 112). During storage on the farm, a decrease in strain diversity and an intensive growth of *LAB* strains, mainly *Limosilactobacillus fermentum*, were observed. A notable higher number of *Hafnia* (*N* = 14) and *Pseudomonas* (*N* = 32) was detected in the tanker samples. As the process advances, especially after pasteurization, a significant reduction in microbial presence is evident, demonstrating the effectiveness of thermal treatment. Only a few genera, such as *Micrococcus* and *Bacillus*, persist through to later stages like crystallization and buttermilk, while no microorganisms were detected in the final product – butter.

**Fig. 5:**
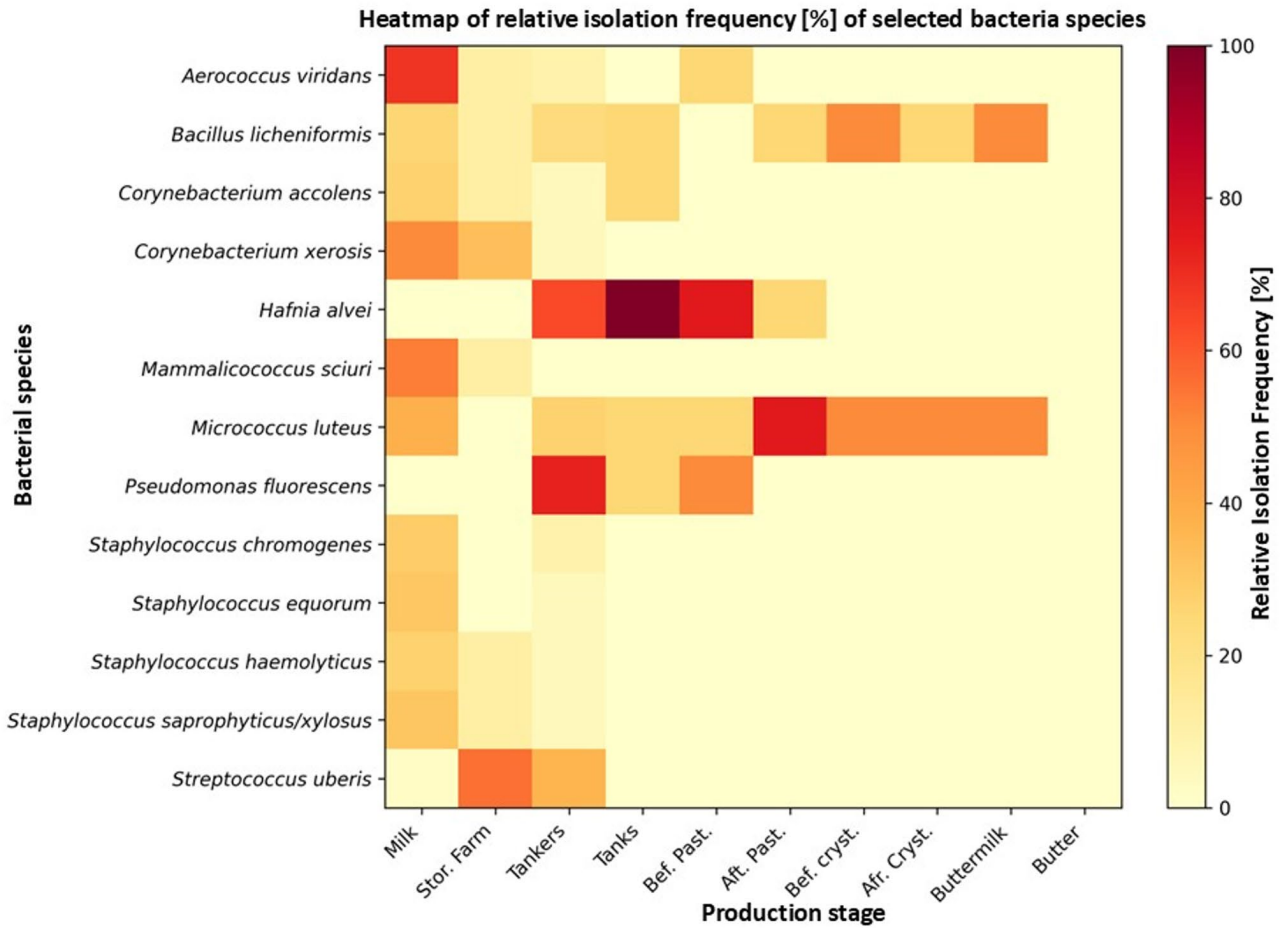
Changes in the frequency of the most prevalent species across different stages of milk storage and processing. The colors on the heat map correspond to the relative abundance of each species at a given processing stage.

The relative abundance of the most prevalent species varied across different stages of milk storage and processing (Fig. 5). The most significant changes were observed for *Bacillus licheniformis* and *Micrococcus luteus*, both of which remained dominant throughout milk processing. During the early stages of storage (from raw milk to storage in tanks), the population of *B. licheniformis* remained relatively stable (ranging from 26% to 11% of samples). However, in subsequent processing stages, its relative abundance increased significantly, reaching 50% before crystallization and 50% in buttermilk. Interestingly, the abundance of *M. luteus* remained unchanged during crystallization, stabilizing at 50% and decreasing to 22% in buttermilk. In addition, the frequency of *M. luteus* occurrence before pasteurization was unchanged, whereas after pasteurization it increased intensively to 75% and remained at the level of 50% of samples in the subsequent stages.

## Discussion

Milk naturally contains a mixture of beneficial probiotic bacteria as well as undesirable psychrotrophic (a major cause of food spoilage) and potentially detrimental species (opportunistic pathogens)^17^. In the microbial cultivation assays, culture conditions were deliberately selected to support the growth of these target organisms, as actively growing and cultivable bacteria are most directly responsible for technological problems during dairy processing, such as rapid milk acidification, biofilm formation, and product spoilage. However, it is important to acknowledge that such conditions may limit the detection of more fastidious or heat-sensitive strains, such as lactic acid bacteria, which are commonly reported in dairy products. In this study, the application of MALDI-TOF MS, due to its speed and cost-effectiveness, enabled the use of multiple culture conditions for individual sample analysis, thereby increasing species coverage and providing identification results comparable to sequencing without the delays associated with traditional culturing. Considering the practical constraints of 16 S rRNA sequencing—its labor-intensive nature and high cost in Poland (approximately $150–200 per sample, versus ~$30 per sample for MALDI-TOF MS for 5–10 isolates), using sequencing for multiple isolates per sample would have been financially prohibitive for routine analysis in the food industry. Therefore, while sequencing-based approaches, such as next-generation sequencing, remain the gold standard for microbiota profiling, yielding the most comprehensive data about the sample, MALDI offers a more practical and versatile approach for routine monitoring across various industrial applications, as it strikes the best compromise between completeness of microbiota deciphering and cost and turnaround time of analysis.

In this study, using MALDI-TOF MS, we identified a total of 146 different microorganisms in raw bovine milk and butter production intermediates, the majority of which were bacteria (139 species). Randomly selected identifications were validated by 16 S rRNA gene sequencing confirming the initial species-level identifications in most cases. Nevertheless, 16 S rRNA gene sequencing has limited discriminatory power for closely related species, particularly within the genera Bacillus and Streptococcus, as also observed in this study. In such cases, whole-genome sequencing (WGS) would allow unequivocal species assignment, while species-specific qPCR assays or amplification and sequencing additional house-keeping genes like *rpoB*, *gyrB*, *atpD*, and *recA* could serve as a rapid confirmatory tool for selected taxa. The continuous development of the MALDI technique expanded its application to environmental samples such as milk, providing high concordance with 16 S rRNA identification^18^.

Our study showed that the most common strains in bovine milk were *Staphylococcus*, *Corynebacterium* and *Aerococcus*. These findings are consistent with recent literature describing the core microbiota composition of bovine milk^19,20^. All these genera are considered to be responsible for milk contamination and represent the most relevant mastitis-related bacteria in dairy farming. Bexiga et al. showed that milk collection with a cannula significantly reduces milk contamination caused by *Corynebacterium* and *Staphylococcus* strains^21^. Bayle et al. research, in turn, demonstrated that the air in milking parlors harbors a diverse range of bacterial genera, notably including *Staphylococcus*, *Bacillus*, and *Aerococcus*, which may contribute to the microbial contamination of milk during the milking process^22^. Differences in udder milk microbiota were observed between the March and April sampling points, coinciding with an increase in average air temperature reported by the Institute of Meteorology and Water Management (from 6.4 to 10.3 °C). In March, *Staphylococcus* spp. and *Mammaliicoccus sciuri* were more frequent, whereas in April higher prevalence of *Aerococcus viridans* and *Corynebacterium xerosis* was noted. These observations are consistent with the results presented by Pałczyńska et al.^23^, which confirms that seasonal fluctuations in ambient temperature significantly modify the composition and distribution of individual bacterial taxa.

Within the genus *Corynebacterium*, 19 different species were identified, most commonly *C. xerosis*, a commensal of mammalian mucosa explaining its frequent isolation from row milk^24^. Large proportion of *Corynebacterium* species, including *C. xerosis*,* C. amycolatum*, *C. pilosum*, may originate from the cattle environment (milking equipment, bedding, floors), with direct transmission during milking playing a major role, highlighting the importance of hygiene^25^. Kirkeby et al. study found that the direct transmission model (e.g. during milking) better describes the spread of these bacteria than the environmental transmission model, which highlights the importance of hygienic practices during milking to limit their spread in the herd^26^. Some of identified bacteria, *C. amycolatum* and *C. minutissimum*, are also associated with clinical or subclinical mastitis in cattle^24^, while others, like *C. casei*^27^ and *C. glutamicum*^28^ play beneficial roles in food processing.

In the present study, the most frequently identified *Staphylococcus* species in bovine milk belonged to the Coagulase-Negative Staphylococci (CNS) group, including *Mammaliicoccus sciuri* (53% samples), *S. xylosus/saprophyticus* (31%), *S. chromogenes* (29%), and *S. haemolyticus* (27%). These species are common in bovine milk, acting as commensals or opportunistic pathogens often linked to subclinical forms of mastitis^29^. Notably, *S. xylosus*, *S. chromogenes*, and *S. haemolyticus* species have been reported for some time as dominant in the bovine milk microbiota in Poland, further confirming their prevalence and relevance in the local dairy context^23,30,31^. *M. sciuri* occurred significantly more often (*p* ≤ 0.05) in milk from hind quarters, likely due to greater contamination exposure of the hind teats. The presence of *M. sciuri* strains may considerably increase the risk of recalcitrant infections, owing to their potential role as reservoirs of antibiotic resistance genes, which can be horizontally transferred to more virulent pathogens such as *S. aureus*^32^. Although *S. aureus* was not detected, several Gram-negative mastitis-associated bacteria were present. The most frequently was *Escherichia coli −* 9% of the analyzed samples. Other genera were identified less frequently, including *Pseudomonas*,* Raoultella*,* Citrobacter*,* Proteus*,* Klebsiella*, and *Serratia*. The presence of *E. coli* is globally recognized as the main pathogen during mastitis infection and its presence indicates poor hygiene^33^. The prevalence of udder-infecting strains may reflect closed herd management, which limits external pathogens but allows persistence of resident bacteria. Sjostrom et al. demonstrated that, despite indoor cows exhibiting better hygiene scores compared to outdoor cows, they experienced a higher incidence of clinical mastitis^34^.

In the vast majority of samples the presence of bacteria of the genus *Aerococcus* was detected, especially *A. viridans* (69% of samples), less frequently *A. urinaeequi* (15%). Their presence in milk is primarily attributed to environmental contamination, particularly from fecal matter, manure, or bedding materials commonly used in barns^35,36^. This means they can be found in almost all cattle populations. Their high presence in the milk of healthy animals in this study primarily indicates contamination during milking. Recently, *A. viridans* in raw milk is gaining attention as an emerging mastitis-associated microbe, reducing milk yield, altering composition, and increasing somatic cell counts^37,38^. In turn, recent scientific reports indicate that the presence of *A. urinaeequi* strains may exert a beneficial influence on the milk microbiota by inhibiting the colonization of opportunistic pathogens such as *E. coli*^39^. In this study, in most samples where *A. urinaeequi* strains were present, no pathogenic Gram-negative bacteria growth were noted.

Among the bacteria naturally occurring in bovine milk but not typically detected in the farm environment, potentially beneficial bacteria of the genera *Lactococcus*, *Lactobacillus*,* Leuconostoc*, and some *Streptococcus* are often mentioned, and their abundance may vary^17,40^. Their presence in this study was detected in only a few samples of milk, which was likely due to their sensitivity to environmental conditions, combined with high animal density and closed breeding of the herd. Many studies indicate that milking practices and udder disinfectants used, as well as the type of milking parlor and the presence of hay in the bedding area significantly influence changes in the content of LAB strains^41,42^. LAB have the ability to grow and be metabolically active over a wide temperature range, including the low temperatures typical of dairy refrigeration plants, which confirms our observations^43^. Low-temperature milk storage increased the detectability of LAB strains in milk, particularly *Limosilactobacillus fermentum*, which was often isolated from samples from the farm tank and at later stages of production. An important issue that should also be considered is the cultivation of microorganisms. Using different cultivation conditions, more favourable for the growth of LAB species, could increase their detection in this study.

The presence of *Bacillus* strains in milk is important due to their persistence in dairy environments and association with contamination. In this study, the main presence of *B. licheniformis* (26% of samples), *B. pumilis* (14%) and *B. subtilis* group (9%) was noted. Even at low levels in raw milk should be carefully monitored, as they pose risks to product quality and overall hygiene^44^. Our study confirms that although *Bacillus* bacteria were identified in relatively low numbers in fresh milk, their prevalence increased at later stages of production, including post-pasteurization. This may reflect heat activation of bacterial spores, where pasteurization inadvertently triggers the germination of heat-stable bacterial spores by disrupting their protective layers, triggering germination and subsequent growth in milk. It mainly applies to the sublethal temperatures of pasteurization (72–76 °C); even higher temperatures (> 80 °C), can allow some damaged spores to recover and outgrow, but in the longer timeline (during storage)^45^. Certain *Bacillus* species, including *B. cereus* and *B. licheniformis*, survive pasteurization and UHT treatments, adhere to surfaces, form biofilms, and may proliferate in milk or cream^46^. In our study, *B. licheniformis*, capable of strongly adhering and forming biofilms on stainless steel^47^, survived the pasteurization process of cream at 92 °C for about 30–40 s and was finally detected in buttermilk. Earlier research has demonstrated a widespread presence of this bacteria across various dairy products, including powdered infant formula, non-fat dry milk powders, UHT milk, pasteurized milk, and cheese^48,49^. Some strains of *B. licheniformis* have probiotic properties and beneficial effects on milk composition and flavor^46^. Lamontagne et al. demonstrated that orally supplementing cows with *B. subtilis* and *B. licheniformis* bacteria can positively influence both milk yield and the fatty acid composition of the milk^50^. However, it is important to note that certain isolates of *B. licheniformis* produce enterotoxins or heat-resistant enzymes, contributing to spoilage and potential foodborne illness, highlighting the need for careful monitoring throughout dairy production^12,51^.

Low temperatures used for preserving milk before processing are necessary to inhibit the growth of mesophilic pathogens, but shift the microbiota of raw milk towards psychrotrophic bacteria. In samples from tanks transporting milk to the dairy and initial processing stages, intensive growth of psychrotrophic bacteria belonging to the genera *Pseudomonas*,* Hafnia*,* Rauoltella*,* Serratia*, and *Acinetobacter* was observed. This issue has also been confirmed by other studies. In previous studies^52^, *Pseudomonas fluorescens* was the most frequently identified microorganism in refrigerated milk samples. Research indicates that 3–4 days of milk storage increases the abundance of *Pseudomonas* and *Acinetobacter*^53^, accordingly, their increase was observed from the storage stage onward in our study. Of particular significance in hygiene is the capacity of these bacteria to produce extracellular biopolymers, facilitating their permanent attachment to surfaces and biofilm formation, both in monocultures and complex microbial communities. These biofilms pose a serious hygiene hazard, are difficult to remove using traditional cleaning methods, and their presence favors secondary contamination of milk, even after pasteurization^54^. In this regard, it is pertinent to note that dairy tanks are cleaned after each milk batch before refilling, indicating potential procedural inadequacies in this study. Furthermore, their presence in milk processing is problematic due to rapid growth at low temperatures and the secretion of thermostable enzymes that can result in spoilage, thus long-term refrigerated storage of milk is discouraged^55^. During milk transport to pasteurization, significant *Hafnia alvei* proliferation was observed. Although it is not classified as a typical pathogen, its presence suggests inadequate hygiene (also biofilm-forming) and may adversely influence the shelf life and sensory properties of dairy products due to enzyme activity^56^.

*Micrococcus luteus* was the sole species found in all analyzed samples except butter. This bacterium contaminates milk through various sources, including udder skin and equipment. It demonstrates resilience to harsh environmental conditions, facilitating its survival in dairy environments. Certain strains may possess antibiotic resistance and form biofilms, complicating control measures^57^. While not pathogenic, its presence can induce undesirable changes in milk quality and organoleptic changes during prolonged storage. In this study, environmental samples were not collected from the barn and dairy. However, the literature indicates that *M. luteus* is a well-known airborne bacterium and a frequent source of contamination^58^.

Butter was the only product analyzed in which no microbial growth was detected, attributed to its high fat content, low water activity, and hygienic packaging. Sample preparation may have influenced the results to some extent. In this study, the butter samples were first incorporated into a warm liquid medium to facilitate dissolution, which could have favored the growth of thermophilic organisms. However, this method may have hindered the detection of sensitive strains like LAB, commonly found in similar products^59^. Nevertheless, the species identified in earlier stages as potentially hazardous to consumers, particularly *B. licheniformis*, are known to be heat-resistant, and their presence was not detected in butter.

However, in buttermilk samples, despite its acidic pH, the presence of various species of environmental bacteria was detected. Considering that buttermilk is often the starting raw material for the production of kefir and other fermented milk products, the presence of these microorganisms can pose a significant threat to the quality and safety of the final product. Therefore, it is necessary to systematically improve production processes and tighten hygiene procedures at all stages of production to ensure optimal microbiological purity and prevent the risk of microbiological contamination.

## Conclusion

This study revealed key changes in microbial diversity during butter production in a Polish dairy, associated with processing steps such as milk storage and pasteurization. The initial microbiological quality of raw cow’s milk is crucial for the quality and safety of intermediate products, representing a major source of microorganisms in the production chain. This highlights the importance of hygiene, animal health, and milking practices in controlling microbial load at the beginning of the process. Applying the MALDI identification method demonstrates a feasible approach for systematic control of this complex production chain. As our study showed, the use of the rapid MALDI identification together with multiple culture conditions enabled pinpointing the tankers as a key microbial contamination hotspot in the investigated dairy plant, mainly as a result of suboptimal cleaning of transport equipment or fluctuations in temperature during milk transfer, which can create favorable conditions for bacterial growth.

Moreover, applying a structured sampling and analysis protocol allowed us to evaluate the effectiveness of the manufacturer’s thermization and pasteurization processes, as well as to monitor microbial contamination in the post-pasteurization environment of the dairy plant. The obtained results showed that although pasteurization conditions reduced microbial richness, eliminating the majority of bacteria and fungi, the presence of specific spore-forming or heat-tolerant species in samples collected after pasteurization indicated a possible risk of recontamination from survival niches and/or the possibility of heat-activated spore germination phenomena.

Summarizing, the implementation of fast MALDI identification coupled with multiple culture conditions demonstrates the promising strategy to expand the feasibility of the traditional microbial culturing methods. By enabling the detection and identification of a broader range of microorganisms than those typically targeted by routine quality standards (such as total bacterial count, *E. coli*, *S. aureus*, *Listeria monocytogenes*, *Salmonella* spp.) this approach not only improves the efficiency and comprehensiveness of species-level identification but also enhances the monitoring of microbial safety and hygiene in dairy production. In this mode, MALDI provides a versatile tool for industrial applications, enabling timely detection of potential contaminants, assessment of cleaning and sanitation effectiveness, and proactive management of microbial risks across the production chain.

## Supplementary Information

Below is the link to the electronic supplementary material.

Supplementary Material 1

Supplementary Material 2

Supplementary Material 3

## Data Availability

All sequence data that support the findings of thisstudy have been deposited in GenBank and the public URL for the sequences collection is [https://www.ncbi.nlm.nih.gov/sites/myncbi/1F1i8raBplj5d/collections/66707925/public/](https:/www.ncbi.nlm.nih.gov/sites/myncbi/1F1i8raBplj5d/collections/66707925/public).
