# Supplementary material for: Application of MALDI-TOF-MS in the surveillance of microbial diversity in butter production: a case study of Polish dairy
Source: Sci Rep. 2026 Mar 11;16:13092. doi: 10.1038/s41598-026-43570-0 (PMC13099988; doi:10.1038/s41598-026-43570-0)
Supplement: Supplementary file 1 [file 41598_2026_43570_MOESM1_ESM.odt]

Supplementary Table S1.  The number of identified microorganisms (N) in individual milk processing stages. R. Milk- raw milk, Tankers - cold store at the farmer's, Tank - storage in a tank at the dairy, Bef.Past- before pasteurization, After.Past- after pasteurization, Bef.Cryst- before crystallization, After.Cryst- after crystallization.

| Milk processing stages | Identified species/genus | N |
| --- | --- | --- |
| Buttermilk | Bacillus cereus group | 1 |
| Bacillus licheniformis | 2 |
| Enterococcus faecium | 2 |
| Micrococcus luteus | 2 |
| Moraxella osloensis | 1 |
| Priestria megaterium | 1 |
| N total | 9 |
| Stor.Farm. Stor.Farm. | Acinetobacter pseudolwoffii | 1 |
| Aerococcus viridans | 1 |
| Bacillus licheniformis | 1 |
| Bacillus pumilis | 1 |
| Brucella anthropi | 1 |
| Candida tropicalis | 1 |
| Chryseobacterium hominis | 1 |
| Corynebacterium accolens | 1 |
| Corynebacterium amycolatum | 1 |
| Corynebacterium xerosis | 3 |
| Enterococcus faecium | 1 |
| Escherichia coli | 3 |
| Kocuria kristinae | 1 |
| Kocuria salsicia | 3 |
| Kocuria varians | 3 |
| Lactobacillus delbrueckii | 3 |
| Lactobacillus fermentum | 3 |
| Lactobacillus rhamnosus | 3 |
| Mammaliicoccus sciuri | 1 |
| Micrococcus luteus | 1 |
| Moraxella osloensis | 1 |
| Pasturella multocida | 3 |
| Pichia | 1 |
| Rothia endophytica | 1 |
| Staphylococcus cohnii | 1 |
| Staphylococcus epidermidis | 1 |
| Staphylococcus gallinarum | 1 |
| Staphylococcus haemolyticus | 1 |
| Staphylococcus succinus | 1 |
| Staphylococcus xylosus | 1 |
| Streptococcus salivarius/vestibularis | 2 |
| Streptococcus uberis | 5 |
| Trichosporon | 1 |
| Trueperella pyogenes | 1 |
| Vanrija humicola | 1 |
| N total | 56 |
| After.Cryst. | Bacillus licheniformis | 1 |
| Micrococcus luteus | 2 |
| Staphylococcus capitis | 2 |
| N total | 5 |
| After.Past. | Alkalihalobacillus clausii | 1 |
| Bacillus licheniformis | 1 |
| Exiguobacterium profundum/aestuartii | 1 |
| Hafnia alvei | 1 |
| Kluyveromyces marxianus | 1 |
| Micrococcus luteus | 3 |
| Staphylococcus epidermidis | 1 |
| Staphylococcus hominis | 1 |
| N total | 10 |
| Bef.Cryst. | Bacillus licheniformis | 2 |
| Kocuria varians | 1 |
| Micrococcus luteus | 2 |
| N total | 5 |
| Bef.Past. Bef.Past. | Acinetobacter lwoffii | 1 |
| Aerococcus viridans | 1 |
| Aeromonas caviae | 1 |
| Aeromonas hydrophila | 1 |
| Buttauxella | 2 |
| Candida inconspicua | 3 |
| Citrobacter freudnii | 1 |
| Corynebacterium amycolatum | 1 |
| Corynebacterium favescens | 1 |
| Corynebacterium flavescens | 1 |
| Escherichia coli | 1 |
| Hafnia alvei | 3 |
| Klebsiella pneumoniae | 1 |
| Kluyveromyces marxianus | 1 |
| Lactobacillus fermentum | 2 |
| Lactococcus garvieae | 1 |
| Lactococcus lactis | 1 |
| Lelliottia amnigena | 1 |
| Leuconostoc mesenteroides | 2 |
| Lysinibacillus | 1 |
| Micrococcus luteus | 1 |
| Pantoea agglomerans | 1 |
| Pichia | 1 |
| Pseudomonas | 2 |
| Pseudomonas fluorescens | 2 |
| Raoultella ornithinolytica | 2 |
| Serratia liquefaciens | 1 |
| Staphylococcus epiderminis | 1 |
| Streptococcus | 1 |
| Streptococcus agalactiae | 1 |
| N total | 40 |
| R.Milk R.Milk R.Milk R.Milk R.Milk R.Milk | Acinetobacter | 13 |
| Acinetobacter baumannii | 3 |
| Acinetobacter bereziniae | 1 |
| Acinetobacter pseudolwoffi | 1 |
| Acinetobacter pseudolwoffii | 1 |
| Acinetobacter towneri | 2 |
| Acinetobacter vivianii | 1 |
| Acinomyces | 1 |
| Actinomyces | 1 |
| Actinomyces oris | 3 |
| Aerococcus | 1 |
| Aerococcus urinaeequi | 33 |
| aerococcus viridans | 1 |
| Aerococcus viridans | 197 |
| Aeromonas hydrophila | 1 |
| Alkalihalobacillus clausii | 4 |
| Aspergillus | 1 |
| Bacillus | 7 |
| bacillus atrophaeus | 1 |
| Bacillus atrophaeus | 1 |
| Bacillus cereus group | 7 |
| bacillus licheniformis | 2 |
| Bacillus licheniformis | 51 |
| Bacillus pumilis | 26 |
| Bacillus simplex | 2 |
| Bacillus subtilis group | 15 |
| bacillus subtilis group group | 1 |
| Bacillus subtilis group group | 4 |
| Bacteroides | 2 |
| Brevibacterium | 3 |
| Brevibacterium linens | 1 |
| Brevundimonas diminuta | 1 |
| Candida | 4 |
| Candida krusei | 8 |
| Candida lambica | 1 |
| Candida tropicalis | 1 |
| Capnocytophaga | 1 |
| Citrobacter freundii | 1 |
| Comamonas | 1 |
| Comamonas kerstersii | 1 |
| Corynebacterium | 167 |
| Corynebacterium accolens | 69 |
| Corynebacterium afermentans | 1 |
| Corynebacterium amycolatum | 20 |
| Corynebacterium argentoratense | 1 |
| Corynebacterium aurimucosum | 1 |
| Corynebacterium callunae | 1 |
| Corynebacterium camporealensis | 1 |
| Corynebacterium casei | 30 |
| Corynebacterium confusum | 1 |
| Corynebacterium flava/flavescens/subflafa/perflava | 1 |
| Corynebacterium freneyi | 31 |
| Corynebacterium glutamicum | 11 |
| Corynebacterium lipophiloflavum | 1 |
| Corynebacterium minutissimum | 27 |
| Corynebacterium pilosum | 28 |
| Corynebacterium striatum | 28 |
| Corynebacterium tuberculostearicum | 4 |
| Corynebacterium viridans | 1 |
| Corynebacterium xerosis | 127 |
| Corynebacterium xylosus | 2 |
| Curtobacterium | 2 |
| Curtobacterium flaccumfaciens | 1 |
| Cutaneotrichosporon | 1 |
| Delftia acidovorans | 2 |
| Diutina rugosa | 1 |
| Empedobacter | 1 |
| Enterococcus | 5 |
| Enterococcus avium | 2 |
| Enterococcus faecium | 7 |
| Enterococcus hirae | 1 |
| Enterococcus malodoratus | 2 |
| Enterococcus raffinosus | 3 |
| Escherichia  coli | 6 |
| Escherichia coli | 16 |
| Exiguobacterium | 1 |
| Exiguobacterium profundum/aestuartii | 2 |
| Facklamia | 1 |
| Gemella haemolysans | 1 |
| Globicatella | 2 |
| Globicatella sanguinis | 6 |
| Glutamicibacter | 2 |
| Klebsiella pneumonaie | 1 |
| Kluyveomyces marxianus | 2 |
| Kluyveromyces marxianus | 1 |
| Kocuria | 2 |
| Kocuria  rhizophila | 3 |
| Kocuria rhizophila | 5 |
| Lactobacillus | 5 |
| Lactobacillus fermentum | 1 |
| Lactococcus garvieae | 1 |
| Listeria | 1 |
| Lysinibacillus | 2 |
| Mammaliicoccus sciuri | 140 |
| Microbacterium | 1 |
| Micrococcus | 1 |
| Micrococcus endophyticus | 2 |
| Micrococcus luteus | 98 |
| Moraxella osloensis | 2 |
| Mycobacterium | 2 |
| Naganishia | 2 |
| Neisseria | 1 |
| Neisseria  mucosa/macacae/sicca | 1 |
| Neisseria flava/flavescens/subflafa/perflava | 1 |
| Neisseria mucosa/macacae/sicca | 2 |
| Oligella | 2 |
| Paenibacillus | 7 |
| Paenibacillus amycolyticus | 1 |
| paenibacillus amylolyticus | 1 |
| Paenibacillus lautus | 2 |
| Paenibacillus macerans | 2 |
| Pantoea agglomerans | 1 |
| Penicillium | 1 |
| Penicillum | 2 |
| Penicillum oxalicum | 1 |
| Pichia | 1 |
| Priestria megaterium | 2 |
| Proteus mirabilis | 2 |
| Proteus vulgaris | 2 |
| Pseudomonas | 4 |
| Pseudomonas mendocina | 1 |
| Pseudomonas stutzeri | 1 |
| Psychrobacter | 3 |
| Psychrobacter pulmonis | 1 |
| Psychrobacter sanguinis | 8 |
| Raoultella ornithinolytica | 4 |
| Rhodococcus | 2 |
| Rothia | 6 |
| Rothia aeria | 2 |
| Rothia dentocariosa | 1 |
| Rothia endophytica | 2 |
| Schaalia | 2 |
| Serratia liqefaciens | 1 |
| Serratia marcescens | 3 |
| Serratia proteamaculans | 1 |
| Sphingobacterium | 2 |
| Sphingobacterium mizutaii | 1 |
| Sphingomonas | 2 |
| Staphylococcus | 31 |
| Staphylococcus auricularis | 1 |
| Staphylococcus borealis | 1 |
| Staphylococcus capitis | 5 |
| Staphylococcus chromogenes | 77 |
| Staphylococcus cohnii | 6 |
| Staphylococcus dysgalactiae | 1 |
| Staphylococcus epidermidis | 12 |
| Staphylococcus equorum | 74 |
| Staphylococcus gallinarum | 10 |
| Staphylococcus haemolyticus | 58 |
| Staphylococcus homini | 1 |
| Staphylococcus hominis | 12 |
| Staphylococcus intermedius | 2 |
| Staphylococcus lutetiensis | 1 |
| Staphylococcus petrasii | 4 |
| Staphylococcus saprophiticus | 1 |
| Staphylococcus succinus | 35 |
| Staphylococcus warneri | 5 |
| Staphylococcus xerosis | 1 |
| Staphylococcus xylosus | 64 |
| Stenotophomonas acidaminiphila | 1 |
| Streptococcus | 4 |
| Streptococcus agalactiae | 2 |
| Streptococcus dysgalactiae | 4 |
| Streptococcus henryi | 1 |
| Streptococcus lutetiensis | 3 |
| Streptococcus mitis/oralis | 6 |
| Streptococcus peroris | 1 |
| Streptococcus pseudopneumoniae | 1 |
| Streptococcus pyogenes | 1 |
| Streptococcus salivarius/vestibularis | 3 |
| Streptococcus sanguinis | 6 |
| Streptococcus uberis | 4 |
| Streptomyces | 1 |
| Trichosporon | 1 |
| Turicella | 2 |
| Vanrija | 1 |
| Vanrja humicola | 1 |
| Weissella  pamesenteroides | 2 |
| N total | 1823 |
| Tank Tank | Aspergillus | 1 |
| Bacillus licheniformis | 1 |
| Bacillus pumilis | 1 |
| Candida | 1 |
| Candida inconspicua | 1 |
| Candida krusei | 1 |
| Chryseobacterium | 1 |
| Corynebacterium accolens | 1 |
| Corynebacterium minutissimum | 1 |
| Escherichia coli | 1 |
| Ewingella | 1 |
| Hafnia alvei | 4 |
| Kocuria rhizophila | 1 |
| Lactobacillus | 1 |
| Lactobacillus fermentum | 1 |
| Lactococcus garvieae | 1 |
| Lactococcus lactis | 1 |
| Leuconostoc mesenteroides | 3 |
| Lysinibacillus | 1 |
| Micrococcus luteus | 1 |
| Moraxella osloensis | 2 |
| Pasturella multocida | 1 |
| Pseudomonas | 1 |
| Pseudomonas azotoformans | 1 |
| Pseudomonas fluorescens | 1 |
| Raoultella ornithinolytica | 1 |
| Staphylococcus aureus | 1 |
| Staphylococcus hominis | 1 |
| Staphylococcus xylosus | 1 |
| Streptococcus dysgalactiae | 1 |
| Streptococcus suis | 1 |
| N total | 37 |
| Tankers Tankers | Acinetobacter johnsonii | 6 |
| Acinetobacter lwoffii | 3 |
| Aerococcus viridans | 2 |
| Aeromonas caviae | 2 |
| Aeromonas hydrophila | 1 |
| Aeromonas rivipollensis | 1 |
| Bacillus | 1 |
| Bacillus licheniformis | 5 |
| Bacillus pumilis | 1 |
| Buttauxella | 2 |
| Candida | 1 |
| Candida inconspicula | 1 |
| Candida krusei | 2 |
| Candida lipolytica | 1 |
| Chryseobacterium | 1 |
| Corynebacterium accolens | 1 |
| Corynebacterium amycolatum | 1 |
| Corynebacterium aurimucosum | 1 |
| Corynebacterium pilosum | 1 |
| Corynebacterium propinquum | 1 |
| Corynebacterium striatum | 2 |
| Corynebacterium tuberculostearicum | 1 |
| Corynebacterium xerosis | 1 |
| Curtobacterium | 1 |
| Diutina rugosa | 1 |
| Enterobacter | 1 |
| Escherichia coli | 4 |
| Gordonia | 1 |
| Hafnia alvei | 14 |
| Kluyveromyces marxianus | 2 |
| Kocuria | 2 |
| Kocuria salsicia | 2 |
| Kocuria varians | 6 |
| Lactobacillus delbrueckii | 1 |
| Lactobacillus fermentum | 5 |
| Lactococcus garvieae | 1 |
| Lactococcus lactis | 12 |
| Lelliottia amnigena | 1 |
| Leuconostoc | 1 |
| Leuconostoc gelidum | 1 |
| Leuconostoc mesenteroides | 5 |
| Magnusiomyces capitatus | 1 |
| Micrococcus luteus | 6 |
| Tankers | Moraxella osloensis | 4 |
| Pantoea agglomerans | 1 |
| Pasturella multocida | 8 |
| Pichia | 1 |
| Pseudomonas aeruginosa | 5 |
| Pseudomonas anguilliseptica | 1 |
| Pseudomonas azotoformans | 1 |
| Pseudomonas azotoformas | 5 |
| Pseudomonas fluorescens | 16 |
| Pseudomonas mexicana | 1 |
| Pseudomonas rivipollensis | 1 |
| Pseudomonas stutzeri | 1 |
| Pseudomonas veronii | 1 |
| Raoultella terrigena | 2 |
| Serratia liquefaciens | 3 |
| Staphylococcus chromogenes | 2 |
| Staphylococcus cohnii | 1 |
| Staphylococcus epidermidis | 1 |
| Staphylococcus equorum | 1 |
| Staphylococcus gallinarum | 1 |
| Staphylococcus haemolyticus | 1 |
| Staphylococcus succinus | 2 |
| Staphylococcus xylosus | 1 |
| Streptococcus | 1 |
| Streptococcus agalactiae | 2 |
| Streptococcus dysgalactiae | 3 |
| Streptococcus halichoeri | 1 |
| Streptococcus luteiensis | 1 |
| Streptococcus salivarius/vestibularis | 4 |
| Streptococcus sanguinis | 1 |
| Streptococcus suis | 4 |
| Streptococcus uberis | 8 |
| Vanrija humicola | 1 |
| N total | 194 |
| N total at all stages | 2179 |
