# Supplementary material for: Application of MALDI-TOF-MS in the surveillance of microbial diversity in butter production: a case study of Polish dairy
Source: Sci Rep. 2026 Mar 11;16:13092. doi: 10.1038/s41598-026-43570-0 (PMC13099988; doi:10.1038/s41598-026-43570-0)
Supplement: Supplementary file 2 [file 41598_2026_43570_MOESM2_ESM.odt]

Suplementary TableS3. Frequency of occurrence of all species identified using the MALDI technique in the analyzed samples, with some confirmed by 16S rRNA gene sequencing. The table provides detailed characteristics of the identified species, categorized based on their thermophilic (T) or psychrophilic (P) nature, as well as their ability to form spores (S) or biofilms (B).

|  | Raw milk (n=200) | Butter processing (n=63) | Characteristics |
| --- | --- | --- | --- |
| FUNGI |
| Saccharomycetaceae |
| Candida sp. | 4 | 2 |  |
| Candida inconspicua | - | 5 |  |
| Candida krusei | 7 | 3 |  |
| Candida lambica | 1 | - |  |
| Candida lipolytica | - | 1 |  |
| Candida tropicalis | 1 | 1 |  |
| Kluyveomyces marxianus | 3 | 4 |  |
| Debaryomycetaceae |
| Diutina rugosa | 1 | 1 |  |
| Filobasidiaceae |
| Naganishia sp. | 2 | - |  |
| Pichiaceae |
| Pichia sp. | 1 | 3 |  |
| Trichosporonaceae |
| Vanrja humicola | 2 | 2 |  |
| BACTERIA |
| Actinomycetaceae |
| Actinomyces sp. | 2 | - |  |
| Actinomyces oris | 3 | - |  |
| Schaalia sp. | 1 | - |  |
| Aerococcaceae |
| Aerococcus sp. | 1 | - |  |
| Aerococcus urinaeequi | 29 | - | B |
| Aerococcus viridans | 137 | 4 | B |
| Globicatella sanguinis | 6 | - |  |
| Trueperella pyogenes | - | 1 |  |
| Aeromonadaceae |
| Aeromonas caviae | - | 3 |  |
| Aeromonas salmonicida | 1 | 2 |  |
| Aeromonas rivipollensis | - | 2 |  |
| Bicatella sp. | 2 | - |  |
| Facklamia sp. | 1 | - |  |
| Bacillaceae |
| Bacillus sp. | 7 | 1 |  |
| Bacillus atrophaeus | 2 | - |  |
| Bacillus cereus group | 7 | 1 |  |
| Bacillus licheniformis | 51 | 13 | T S B |
| Bacillus pumilis | 25 | 3 | T S B |
| Bacillus simplex | 2 | - |  |
| Bacillus subtilis group | 18 | - | T S B |
| Exiguobacterium sp. | 1 | - |  |
| Exiguobacterium profundum/aestuartii | 2 | 1 |  |
| Lysinibacillus sp. | 2 | 2 |  |
| Priestria megaterium | 2 | 1 |  |
| Shouchella clausii | 4 | - |  |
| Bacteroidaceae |
| Bacteroides sp. | 2 | - |  |
| Brevibacteriaceae |
| Brevibacterium sp. | 3 | - |  |
| Brevibacterium linens | 1 | - |  |
| Brucellaceae |
| Brucella anthropi | - | 1 |  |
| Caulobacteraceae |
| Brevundimonas diminuta | 1 | - |  |
| Comamonadaceae |
| Comamonas kerstersii | 1 | - |  |
| Delftia acidovorans | 2 | - |  |
| Corynebacteriaceae |
| Corynebacterium sp. | 126 | - | B |
| Corynebacterium accolens | 53 | 3 | B |
| Corynebacterium afermentans | 1 | - |  |
| Corynebacterium amycolatum | 19 | 4 | B |
| Corynebacterium argentoratense | 1 | - |  |
| Corynebacterium aurimucosum | 1 | 1 |  |
| Corynebacterium callunae | 1 | - |  |
| Corynebacterium camporealensis | 1 | - |  |
| Corynebacterium casei | 27 | - | B |
| Corynebacterium confusum | 1 | - |  |
| Corynebacterium favescens | - | 2 |  |
| Corynebacterium flava/flavescens/subflafa/perflava | 1 | - |  |
| Corynebacterium freneyi | 27 | - | B |
| Corynebacterium glutamicum | 9 | - |  |
| Corynebacterium lipophiloflavum | 1 | - |  |
| Corynebacterium minutissimum | 23 | 1 |  |
| Corynebacterium pilosum | 23 | 1 | B |
| Corynebacterium propinquum | - | 1 |  |
| Corynebacterium striatum | 22 | 2 | B |
| Corynebacterium tuberculostearicum | 4 | 1 |  |
| Corynebacterium xerosis | 100 | 4 | B |
| Enterobacteriaceae |
| Buttauxella sp. | - | 4 |  |
| Citrobacter freundii | 1 | 1 |  |
| Enterobacter sp. | - | 1 |  |
| Escherichia coli | 18 | 9 | B |
| Hafnia alvei/ Obesumbacterium proteus | - | 22 | T B |
| Klebsiella pneumoniae | 1 | 1 |  |
| Lelliottia amnigena | - | 2 |  |
| Proteus mirabilis | 2 | - |  |
| Proteus terrae | 2 | - |  |
| Raoultella ornithinolytica | 4 | 3 |  |
| Raoultella terrigena | - | 2 |  |
| Enterococcaceae |
| Enterococcus sp. | 5 | - |  |
| Enterococcus avium | 2 | - |  |
| Enterococcus faecium | 7 | 3 |  |
| Enterococcus hirae | 1 | - |  |
| Enterococcus malodoratus | 1 | - |  |
| Enterococcus raffinosus | 3 | - |  |
| Erwiniaceae |
| Pantoea agglomerans | 1 | 2 |  |
| Gordoniaceae |
| Gordonia sp. | - | 1 |  |
| Lactobacillaceae |
| Lactobacillus sp. | 5 | 1 |  |
| Lactobacillus delbrueckii | - | 4 |  |
| Lactobacillus fermentum | 1 | 11 | B |
| Lactobacillus rhamnosus | - | 3 |  |
| Leuconostoc sp. | - | 1 |  |
| Leuconostoc gelidum | - | 1 |  |
| Leuconostoc mesenteroides | - | 10 | P B |
| Weissella pamesenteroides | 1 | - |  |
| Microbacteriaceae |
| Curtobacteriuexm sp. | 2 | 1 |  |
| Curtobacterium flaccumfaciens | 1 | - |  |
| Microbacterium sp. | 1 | - |  |
| Micrococcaceae |
| Glutamicibacter sp. | 2 | - |  |
| Kocuria sp. | 2 | 2 |  |
| Kocuria kristinae | - | 1 |  |
| Kocuria rhizophila | 8 | 1 |  |
| Kocuria salsicia | - | 5 |  |
| Kocuria varians | - | 10 | P B |
| Micrococcus sp. | 1 | - |  |
| Micrococcus endophyticus | 2 | - |  |
| Micrococcus luteus | 76 | 18 | P B |
| Rothia sp. | 6 | - |  |
| Rothia aeria | 2 | - |  |
| Rothia dentocariosa | 1 | - |  |
| Rothia endophytica | 2 | 1 |  |
| Moraxellaceae |
| Acinetobacter sp. | 12 | - | P B |
| Acinetobacter baumannii | 3 | - |  |
| Acinetobacter bereziniae | 1 |  |  |
| Acinetobacter johnsonii | - | 6 |  |
| Acinetobacter guillouiae | - | 4 |  |
| Acinetobacter pseudolwoffi | 2 | 1 |  |
| Acinetobacter towneri | 2 | - |  |
| Acinetobacter vivianii | 1 | - |  |
| Moraxella osloensis | 2 | 8 | P B |
| Psychrobacter sp. | 3 | - |  |
| Psychrobacter pulmonis | 1 | - |  |
| Psychrobacter sanguinis | 8 | - |  |
| Mycobacteriaceae |
| Mycobacterium sp. | 2 | - |  |
| Neisseriaceae |
| Neisseria sp. | 1 | - |  |
| Neisseria flava/flavescens/subflafa/perflava | 1 | - |  |
| Neisseria mucosa/macacae/sicca | 2 | - |  |
| Nocardiaceae |
| Rhodococcus sp. | 2 | - |  |
| Paenibacillaceae |
| Paenibacillus sp. | 7 | - |  |
| Paenibacillus amycolyticus | 2 | - |  |
| Paenibacillus lactis | 2 | - |  |
| Paenibacillus macerans | 2 | - |  |
| Pasteurellaceae |
| Pasturella multocida | - | 12 |  |
| Pseudomonadaceae |
| Pseudomonas sp. | 4 | 3 |  |
| Pseudomonas aeruginosa | - | 5 |  |
| Pseudomonas anguilliseptica | - | 1 |  |
| Pseudomonas azotoformans | - | 7 |  |
| Pseudomonas fluorescens | - | 19 | T P B |
| Pseudomonas mendocina | 1 | - |  |
| Pseudomonas mexicana | - | 1 |  |
| Pseudomonas qingdaonensis | - | 1 |  |
| Pseudomonas stutzeri | 1 | 1 |  |
| Pseudomonas weihenstephanensis | - | 1 |  |
| Pseudomonas veronii | - | 1 |  |
| Staphylococcaceae |
| Gemella haemolysans | 1 | - |  |
| Mammaliicoccus sciuri | 106 | 1 | B |
| Staphylococcus sp. | 31 | - |  |
| Staphylococcus aureus | - | 1 |  |
| Staphylococcus auricularis | 1 | - |  |
| Staphylococcus capitis | 5 | 2 |  |
| Staphylococcus chromogenes | 58 | 2 | B |
| Staphylococcus cohnii | 7 | 2 |  |
| Staphylococcus dysgalactiae | 1 | - |  |
| Staphylococcus epidermidis | 11 | 4 | B |
| Staphylococcus equorum | 61 | 1 | B |
| Staphylococcus gallinarum | 10 | 2 | B |
| Staphylococcus haemolyticus | 54 | 2 | B |
| Staphylococcus hominis | 12 | 2 | B |
| Staphylococcus intermedius | 2 | - |  |
| Staphylococcus petrasii | 4 | - |  |
| Staphylococcus succinus | 30 | 3 | B |
| Staphylococcus warneri | 5 | - |  |
| Staphylococcus xerosis | 1 | - |  |
| Staphylococcus saprophyticus/xylosus | 61 | 3 | B |
| Streptococcaceae |
| Lactococcus garvieae | 1 | 3 |  |
| Lactococcus lactis | - | 14 | B |
| Streptococcus sp. | 4 | 2 |  |
| Streptococcus agalactiae | 2 | 3 |  |
| Streptococcus dysgalactiae | 3 | 4 |  |
| Streptococcus halichoeri | - | 1 |  |
| Streptococcus henryi | 1 | - |  |
| Streptococcus lutetiensis | 4 | 1 |  |
| Streptococcus mitis/oralis | 6 | - |  |
| Streptococcus peroris | 1 | - |  |
| Streptococcus pseudopneumoniae | 1 | - |  |
| Streptococcus pyogenes | 1 | - |  |
| Streptococcus salivarius/vestibularis | 3 | 6 |  |
| Streptococcus sanguinis | 6 | 1 |  |
| Streptococcus suis | - | 5 |  |
| Streptococcus uberis | 4 | 13 | B |
| Streptomycetaceae |
| Streptomyces sp. | 1 | - |  |
| Sphingobacteriaceae |
| Sphingobacterium sp. | 2 | - |  |
| Sphingobacterium mizutaii | 1 | - |  |
| Weeksellaceae |
| Chryseobacterium sp. | - | 2 |  |
| Chryseobacterium hominis | - | 1 |  |
| Yersiniaceae |
| Ewingella sp. | - | 1 |  |
| Serratia liqefaciens | 1 | 4 |  |
| Serratia marcescens | 2 | - |  |
| Serratia proteamaculans | 1 | - |  |
