# Supplementary material for: Application of MALDI-TOF-MS in the surveillance of microbial diversity in butter production: a case study of Polish dairy
Source: Sci Rep. 2026 Mar 11;16:13092. doi: 10.1038/s41598-026-43570-0 (PMC13099988; doi:10.1038/s41598-026-43570-0)
Supplement: Supplementary file 3 [file 41598_2026_43570_MOESM3_ESM.odt]

Suplementary Table S2. Comparison of identification results between the MALDI technique, using two different identification systems, and the 16S rRNA gene sequencing technique. The colors indicate the level of identification consistency: green – identification matched exactly at the species level across all methods, yellow – identification was consistent at the genus level but differed at the species level, blue – the use of 16S rRNA gene sequencing did not allow for obtaining an unambiguous identification, identification within the group/complex.

|  | MALDI-TOF MS |  | 16S rRNA |  |
| --- | --- | --- | --- | --- |
|  | EXS2600 system | Microflex LT system |  | Query cover [%] | Identity [%] |  |
|  | Aerococcaceae |
|  | Aerococcus viridans | 2.34 | Aerococcus sp. | 1.87 | A. viridans | 100 | 99.59 |  |
|  | Globicatella sp. | 1.82 | G. sulfidifaciens | 2.12 | G. sanguinis/ G. sulfidifaciens | 100/ 100 | 99.44/ 99.03 |  |
|  | Aeromonadaceae |
|  | Aeromonas salmonicida | 2.02 | Aeromonas sp. | 1.74 | A. salmonicida | 100 | 99.51 |  |
|  | Aeromonas veronii | 2.25 | Aeromonas sp. | 1.95 | A. rivipollensis | 100 | 99.23 |  |
|  | Bacillaceae |
|  | Bacillus licheniformis | 2.04 | B. licheniformis | 2.02 | B. licheniformis | 100 | 99.31 |  |
|  | Bacillus vallismortis | 2.18 | Bacillus sp. | 1.90 | Bacillus subtilis group (B. amyloliquefaciens/ B. vallismortis) |  | 99.86/ 99.65 |  |
|  | - | <1.7 | - | <1.7 | Bacillus subtilis group (B. vallismortis/ B. subtilis) |  | 99.65/ 99.58 |  |
|  | Bacillus cereus | 2.43 | B. cereus | 2.14 | Bacillus cereus group (B. cereus/ B. tropicus) |  | 99.58/ 99.51 |  |
|  | Bacillus sp. | 1.84 | Bacillus sp. | 1.83 | Bacillus cereus group (B. wiedmannii/ B. toyonensis) |  | 99.38/ 99.38 |  |
|  | - | <1.7 | - | <1.7 | B. stratosphericus/ B. aerius= B. altitudinis |  | 99.43/ 99.37 |  |
|  | Exiguobacterium sp. | 1.86 | Exiguobacterium sp. | 2.03 | E. profundum/ E. aestuarii |  | 98.34/ 98.13 |  |
|  | Priestria megaterium | 2.43 | P. megaterium | 2.20 | P. megaterium | 100 | 99.79 |  |
|  | Shouchella clausii | 2.06 | Shouchella sp. | 1.83 | Shouchella rhizosphaerae |  | 99.51 |  |
|  | Brucellaceae |
|  | Brucella anthropi | 2.20 | Brucella sp. | 1.86 | B. anthropi | 100 | 99.85 |  |
|  | Enterobacteriaceae |
|  | Buttiauxella agrestis | 2.16 | Buttiauxella sp. | 1.94 | Buttiauxella sp. | 100 | 96.05 |  |
|  | Citrobacter freundii | 2.46 | C. freundii | 2.32 | Citrobacter freundii complex (C. freundii/ C. braakii) | 100/ 100 | 98.87/ 98.73 |  |
|  | Escherichia coli | 2.27 | E. coli | 2.35 | E. fergusonii/E. coli |  | 99.44 |  |
|  | Hafnia alvei | 2.41 | H. alvei | 2.39 | H. alvei / Obesumbacterium proteus | 100/ 100 | 99.79/ 99.58 |  |
|  | Klebsiella pneumoniae | 2.30 | K. pneumoniae | 2.27 | K. pneumoniae | 100 | 99.58 |  |
|  | Pantoea agglomerans | 2.27 | P. agglomerans | 2.17 | P. agglomerans | 100 | 99.79 |  |
|  | Proteus vulgaris | 2.01 | Proteus sp. | 1.96 | P. terrae | 100 | 99.65 |  |
|  | Raoultella terrigena | 2.29 | R. terrigena | 2.16 | R. terrigena | 100 | 99.58 |  |
|  | Corynebacteriaceae |  |
|  | Corynebacterium confusum | 2.08 | C. confusum | 2.07 | C.confusum | 100 | 99.78 |  |
|  | Corynebacterium xerosis | 2.09 | C. xerosis | 2.01 | C. xerosis | 100 | 99.35 |  |
|  | Corynebacterium sp. | 1.82 | - | <1.7 | C. casei | 100 | 99.79 |  |
|  | Enterococcaceae |  |
|  | Enterococcus faecium | 2.31 | E. faecium | 2.07 | E. faecium | 100 | 97.50 |  |
|  | Lelliottia amnigena | 2.32 | L. amnigena | 2.17 | L. amnigena | 100 | 99.51 |  |
|  | Micrococcaceae |  |
|  | Kocuria varians | 2.07 | Kocuria sp. | 1.81 | K. varians | 100 | 99.22 |  |
|  | Micrococcus luteus | 2.33 | M. luteus | 2.13 | Micrococcus luteus group (M. yunnanensis/ M. luteus) | 100 | 99.28/ 99.00 |  |
|  | Moraxellaceae |  |
|  | Acinetobacter lwoffii | 2.21 | A. guillouiae | 2.11 | A. guillouiae | 100 | 99.23 |  |
|  | Neisseriaceae |  |
|  | Neisseria mucosa/macacae/sicca | 2.37 | N. macacae | 2.02 | N. macacae | 100 | 99.44 |  |
|  | Paenibacillaceae |  |
|  | Paenibacillus sp. | 1.82 | P. lactis | 2.18 | P. lactis | 100 | 98.82 |  |
|  | Pasteurellaceae |  |
|  | Pasteurella multocida | 2.41 | P. multocida | 2.13 | P. multocida | 100 | 99.79 |  |
|  | Pseudomonadaceae |
|  | Pseudomonas aeruginosa | 2.39 | P. aeruginosa | 2.23 | P. aeruginosa | 100 | 99.93 |  |
|  | Pseudomonas sp. | 1.81 | Pseudomonas sp. | 1.99 | P. weihenstephanensis | 100 | 99.28 |  |
|  | Pseudomonas sp. | 1.90 | - | <1.7 | P. qingdaonensis | 100 | 99.93 |  |
|  | Staphylococcaceae |
|  | Staphylococcus equorum | 2.16 | S. equorum | 2.01 | S. equorum | 100 | 99.79 |  |
|  | Staphylococcus epidermidis | 2.22 | S. epidermidis | 2.00 | S. epidermidis | 100 | 99.38 |  |
|  | Staphylococcus haemolyticus | 2.13 | S. borealis | 2.02 | S. haemolyticus | 100 | 99.79 |  |
|  | Mammaliicoccus sciuri | 2.18 | M. sciuri | 2.01 | M. sciuri | 100 | 99.93 |  |
|  | Staphylococcus warneri | 2.14 | Staphylococcus sp. | 1.86 | S. warneri | 100 | 99.86 |  |
|  | Staphylococcus xylosus | 2.05 | S. xylosus | 2.00 | S. saprophyticus/ S. xylosus | 100/ 100 | 99.59/ 99.38 |  |
|  | Staphylococcus sp. | 1.71 | S. chromogenes | 2.07 | S. chromogenes | 100 | 99.93 |  |
|  | Streptococcaceae |  |
|  | Lactococcus lactis | 2.46 | L. lactis | 2.27 | L. lactis | 100 | 99.58 |  |
|  | Lactococcus garvieae | 2.38 | L. garvieae | 2.30 | L. garvieae | 100 | 99.52 |  |
|  | Streptococcus lutetiensis | 2.12 | S. equinis | 2.01 | S. lutetiensis/equinis | 100 | 99.65 |  |
|  | Streptococcus salivarius | 2.09 | Streptococcus sp. | 1.76 | S. salivarius/ S. vestibularis | 100/ 100 | 99.72/ 99.65 |  |
|  | Streptococcus sanguinis | 2.30 | S. sanguinis | 2.17 | S. sanguinis | 100 | 99.93 |  |
|  | Streptococcus uberis | 2.46 | S. uberis | 2.18 | S. uberis | 100 | 99.79 |  |
| Yersiniaceae |  |  |  |  |  |  |  |
|  | Serratia marcescens | 2.15 | S. marcescens | 2.00 | S. nematodiphila/ S. marcescens | 100/ 100 | 99.58/ 99.30 |  |
